# Fibrinolytic System Changes in Liver Surgery: A Pilot Observational Study

**DOI:** 10.3389/fmed.2018.00253

**Published:** 2018-09-11

**Authors:** Agnese Ozolina, Janis Nemme, Arturs Ozolins, Lars J. Bjertnæs, Indulis Vanags, Janis Gardovskis, Ludmila Viksna, Angelika Krumina

**Affiliations:** ^1^Department of Anesthesiology, Orto Clinic, Riga, Latvia; ^2^Riga Stradins University, Riga, Latvia; ^3^Department of Anesthesiology and Intensive Care Unit, Pauls Stradins Clinical University Hospital, Riga, Latvia; ^4^Department of Surgery, Pauls Stradins Clinical University Hospital, Riga, Latvia; ^5^Anesthesia and Critical Care Research Group, Department of Clinical Medicine, Faculty of Health Sciences, University of Tromsø, The Arctic University of Norway, Tromsø, Norway

**Keywords:** fibrinolysis, liver surgery, tissue plasminogen activator, t-PA, plasminogen activator inhibitor type 1, PAI-1

## Abstract

**Introduction:** Bleeding occurs frequently in liver surgery. Unbalance between tissue plasminogen activator (t-PA) and plasminogen activator inhibitor-1 (PAI-1) concentrations might increase bleeding. Our aim was to analyze perioperative fibrinolytic changes during liver surgery.

**Materials and Methods:** We evaluated 15 patients for inclusion into a prospective pilot study of liver surgery. We assessed fibrinolysis by plasma PAI-1 and t-PA: before surgery (T1), before Pringle maneuver (PM;T2), at the end of surgery (T3) and 24 h postoperatively (T4), and registered demographic and laboratory data, extent and duration of surgery, hemodynamic parameters, blood loss, and transfused volumes of blood products. Data presented as mean ± SD. Significance at *P* < 0.05.

**Results:** After exclusion of six patients only undergoing biopsies, we included six women and three men aged 49.1 ± 19.6 years; two patients with liver metastases of colorectal cancer and hepatocellular carcinoma, respectively, two with focal nodular hyperplasia, two with hepatic hemangioma, and one with angiomyolipoma. Six patients underwent PM. PAI-1 plasma concentration (*n* = 9) rose from 6.25 ± 2.25 at T1 through 17.30 ± 14.59 ng/ml at T2 and 28.74 ± 20.4 (*p* = 0.007) and 22.5 ± 16.0 ng/ml (*p* = 0.04), respectively, at T3 and T4. Correspondingly, t-PA plasma concentration (*n* = 9) increased from 4.76 ± 3.08 ng/ml at T1 through 8.00 ± 5.10 ng/ml (*p* = 0.012) at T2 and decreased to 4.25 ± 2.29 ng/ml and 3.04 ± 3.09 at T3 and T4, respectively. Plasma t-PA level at T2 was significantly different from those at T1, T3, and T4 (*p* < 0.004). In PM patients, t-PA levels increased from T1, peaked at T2 (*p* = 0.001), and subsequently decreased at T3 and T4 (*p* = 0.011 and *p* = 0.037), respectively. Mean blood loss was 1,377.7 ± 1,062.8 ml; seven patients received blood products. Patients with higher PAI-1 levels at T3 received more fresh frozen plasma (*r* = 0.79; *p* = 0.01) and red blood cells (*r* = 0.88; *p* = 0.002).

**Conclusions:** During liver surgery, fibrinolysis increased, as evidenced by rises in plasma PAI-1and t-PA, especially after start of surgery and following PM. Transfused volumes of blood products correlated with higher plasma concentrations of PAI-1. Confirming this tendency requires a larger cohort of patients.

## Introduction

Liver resection is a commonly used procedure for treatment of both benign and malignant hepatic diseases. Although, both anesthetic and surgical techniques have progressed during the last few years, resulting in lower perioperative morbidity, serious complications still occur (1).

The liver is involved in synthesis and clearance of proteins of the fibrinolytic system, which is responsible for the degradation of thrombi in the vasculature (2–4). Fibrinolysis starts with activation of the enzyme tissue plasminogen activator (t-PA), which converts plasminogen to plasmin on the fibrin surface. The concentration of t-PA in blood is regulated by its rate of secretion by endothelial cells, its hepatic clearance, and its inhibition mainly by plasminogen activator inhibitor-1 (PAI-1). It is well established that hyperfibrinolysis occurs in 30–50% of patients undergoing liver surgery (5, 6). Thus, bleeding is one of the most common complications, and it is in most cases unpredicted (7).

To reduce bleeding during liver resection, the surgeon often uses the so-called Pringle maneuver (PM), which consists in clamping the hepatoduodenal ligament, thereby interrupting blood flow through the hepatic artery and portal vein (8). In the case of severe blood losses, blood transfusions may be mandatory. Blood transfusions are associated with a higher mortality and morbidity in patients that are prone to liver resection or other causes of hepatic bleeding (9, 10).

Hepatic bleeding might occur, not only following activation of fibrinolysis, but also secondary to reduced production or rapid consumption of coagulation factors. Hence, limited reserves of coagulation proteins might play a decisive role for the outcome when bleeding occurs in patients with liver diseases (7, 11, 12). However, in liver surgery, it is difficult to sort out whether the bleeding results from changes in hemostasis alone, or to concomitantly occurring hyperfibrinolysis (13–16).

In the present prospective pilot study, we speculate, whether increased fibrinolysis develops secondary to low liver clearance of tissue plasminogen activator (t-PA) in combination with protein C inactivation of PAI-1 (17). These adjustments might promote the conversion of plasminogen into plasmin resulting in a distortion of the balance between t-PA and PAI-1; thus, stimulating increased bleeding during and after liver surgery (1, 7, 18). Changes in the plasma concentrations of PAI-1 and t-PA counted as primary end-points while the associations between the latter, the extent of liver surgery and the amount of transfused blood products served as secondary end-points. Finally, we also aimed at calculating the sample size of a future prospective study of perioperative fibrinolysis in liver surgery.

## Materials and methods

### Population

Fifteen subsequent patients, who were scheduled for liver resection surgery, were included in a prospective pilot observational study in Pauls Stradins Clinical University Hospital in Riga, Latvia. The Medical Research Ethics Committee approved the study protocol and the informed consent form (Approval No 125/28.01.2016).

**Figure 1:**
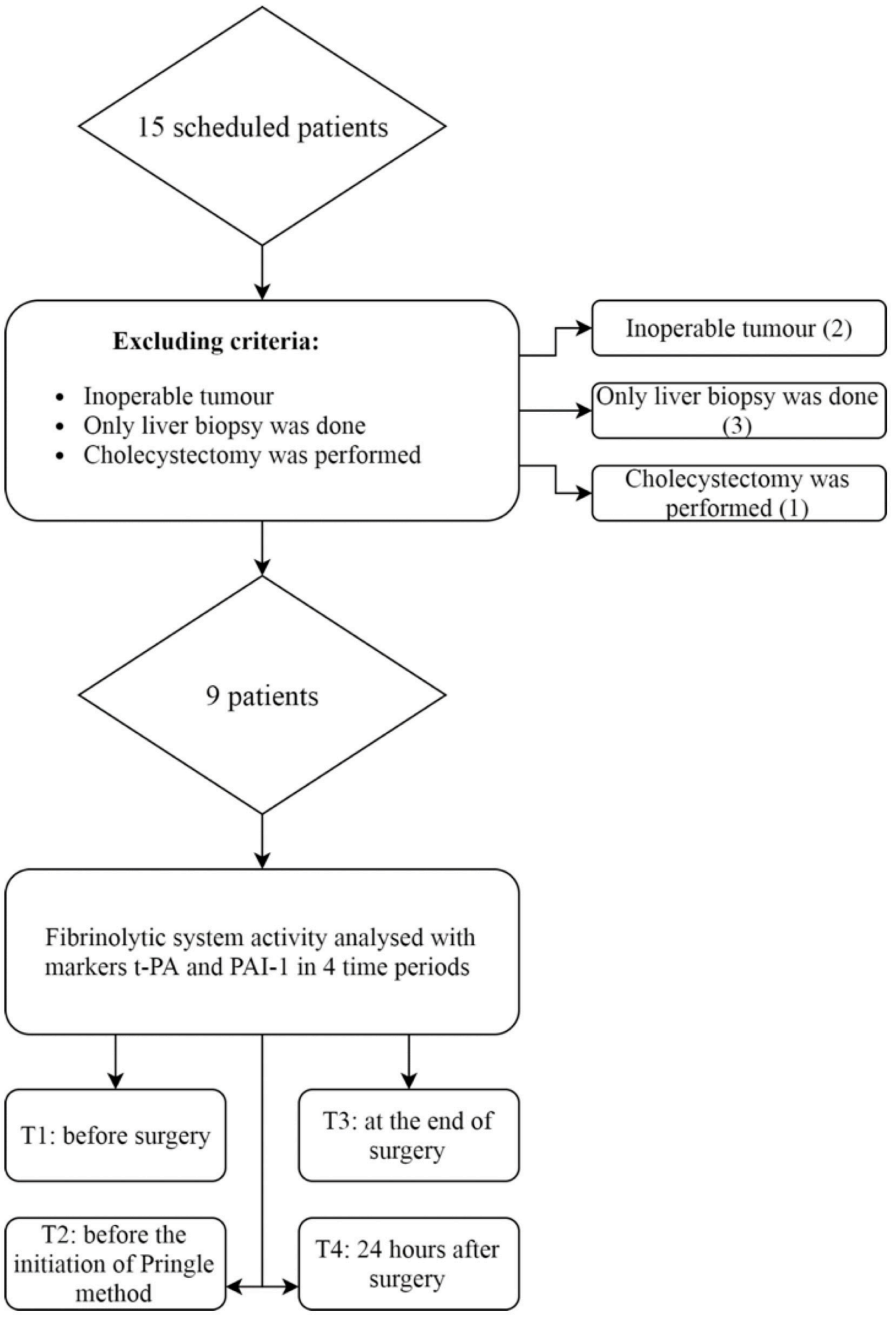
Patients eligible for study of fibrinolysis in liver surgery. Six patients were excluded from the study. Nine patients were analyzed for changes in fibrinolytic markers. t-PA, tissue plasminogen activator; PAI-1, plasminogen activator inhibitor type 1.

We defined the following exclusion criteria: patients < 18 years of age, tumor considered as being radically inoperable or surgery limited to tumor biopsy only (5 patients) or cholecystectomy (1 patient). Therefore, after further analysis, we excluded six patients, as depicted in Figure 1. We obtained informed consent from every patient, or his or hers designated surrogates.

### Perioperative management

All patients underwent standard general anesthesia including endotracheal intubation. We induced anesthesia by injection of midazolam 2.5 mg (Dormicum®, F. Hoffman-La Roche AG, Switzerland), fentanyl (Fentanyl-Kalceks® 0.05 mg/ml, A/S Kalceks, Latvia) 0.01 mg/kg, atracurium (Tracrium® 2 mg/ml, GlaxoSmithKline Manufacturing, S.Polo, Torrile PR, Italy) 0.5 mg/kg and propofol (Propofol-Lipuro® 10 mg/ml, B. Braun Melsungen AG, Germany) 1.5–2 mg/kg intravenously.

Anesthesia was maintained with inhalation of sevoflurane (Sevoflurane®, Piramal Healthcare Ltd, United Kingdom) at 0.8–1.2 MAC, combined with continuous infusion of fentanyl (Fentanyl-Kalceks® 0.05 mg/ml, A/S Kalceks, Latvia) 0.01–0.03 mg/kg/min. Muscle relaxation was obtained with atracurium 0.25 mg/kg per h. All patients received tranexamic acid (TXA) (Amchafibrin® 500 mg/5 ml, Rottapharam S.L., Spain) 10 mg/kg before skin incision. We also provided postoperative epidural analgesia with bupivacaine (Marcain®, AstraZeneca UK Ltd., Macclesfield, Cheshire SK10 2NA UK) 0.5% solution 0.05–0.08 mg/kg at the end of surgery.

On purpose, we accepted relative hypovolemia during surgery, as assessed by keeping the central venous pressure (CVP) between 4 and 8 cmH_2_O to minimize bleeding. If concomitantly, tachycardia (heart rate > 100/min), hypotension (mean blood pressure < 65 mmH_2_O), or oliguria (< 0.5 ml/kg/h) should occur, we administered vasoactive agents, for instance, norepinephrine or dopamine at doses of 0.05–0.2 μg/kg/min and 3–7 μg/kg/min, respectively. Patients, who had blood losses of >10 ml/kg and/or demonstrated hemodynamic instability and signs of severe hypovolemia, including hemoglobin levels < 9 g/L, received blood transfusions.

### Demographic and laboratory data

We collected blood from a peripheral vein into heparin tubes that we cool centrifuged (ELMI CM-6MT®, USA) for 5 min at 3,000 × g. The plasma supernatant was removed from the spun samples and frozen to −70°C until the time of analysis of biomarkers of coagulation and fibrinolysis.

We analyzed fibrinolytic activity at four data collection points by means of the markers PAI-1 and t-PA. Firstly, we determined baseline fibrinolytic activity before surgery (T1). Secondly, we assessed PAI-1 and t-PA plasma concentrations during surgery, before initiation of the Pringle maneuver (T2), in order to evaluate the influence of surgically created tissue damage. Thirdly, we determined the markers at the end of surgery (T3) to assess the impact of liver hypoperfusion on fibrinolytic activity, and fourthly, 24 h after surgery (T4), to detect postoperative fibrinolytic activity, which commonly stays enhanced a few days after liver resection (19).

PAI-1 and t-PA (Technoclone Gmbh®, Vienna Austria) were analyzed with *actibind, an* ELISA method. Plasma concentrations of PAI-1 and t-PA are normally < 20 ng/ml and < 10 ng/ml, respectively.

In addition to PAI-1 and t-PA, we determined the fibrinogen plasma concentration in citrated plasma (Multifibren U reagent, Siemens Healthcare Diagnostics, USA) preoperatively and analyzed prothrombin (PT) with a PT complex assay (Lyophilized Dade® and Innovin®, Siemens Healthcare Diagnostics, USA). We performed all the coagulation tests by means of Sysmex® CA-1500 (Siemens Healthcare Diagnostics, Germany). Hemoglobin (Hb), hematocrit (Hct), platelets, white and red blood cell counts were analyzed by means of a Beckman Coulter LH 750 Hematology Analyzer.

### Statistical analysis

Data were analyzed with SPSS (SPSS® version 20, Chicago, IL) and SigmaPlot (Systat Software, Inc., San Jose, CA), as appropriate. Continuous variables were presented as mean ± standard deviation (SD) or as median and interquartile range; categorical variables as percentages (%). We checked the data for normal distribution with Kolmogorov-Smirnov and Shapiro Wilks tests and used ANOVA for repeated measurements followed by Student-Newman-Keuls *post-hoc* test for all pairwise multiple comparisons of PAI-1 and t-PA vs. time (T1-T4). We used linear regression (Pearson's correlation coefficient) to analyze the relationships between demographic and surgical data as well as PAI-1 and t-PA vs. bleeding volumes. Furthermore, we used Chi-square test to analyze categorical data. We defined a *p* < 0.05 as a statistically significant difference and correspondingly, *p* > 0.05 as not significant (NS). We also calculated sample sizes with comparisons of two means or paired sample *t*-test by using PAI-1 and t-PA data provided α ≤ 0.05 and β ≤ 0.2.

## Results

### Clinical course

Totally 15 consecutive patients underwent liver resection during a 6 months period whereof six patients met exclusion criteria. Table 1 displays baseline demographic characteristics, comorbidities and clinical details, such as the extent and duration of surgery, blood loss, hemodynamic parameters, and volumes of transfused blood products in the remaining nine patients included in the study. We evaluated the patients as belonging to ASA II and III since they all had co-morbidities with different degrees of compensation.

**Table 1:** Baseline demographic characteristics, surgical and hemodynamic parameters.

**Demographic data**	**Totally, *n* = 9 (%)**	**Women, *n* = 6 (%)**	**Men, *n* = 3 (%)**
Age, years	49 ± 19.6	42.5 ± 20.3	62.3 ± 11
Body weight, kg	75 ± 14.5	69 ± 13	87 ± 11.5
**COMORBIDITIES**			
Hypertension, *n* (%)	6 (67%)	3 (50%)	3 (100%)
Diabetes mellitus, *n* (%)	6 (67%)	3 (50%)	3 (100%)
COPD, *n* (%)	2 (22%)	0 (0%)	2 (67%)
**PRIMARY DIAGNOSIS**			
Metastatic colorectal cancer	2 (22%)	1 (17%)	1 (33%)
Hepatocellular carcinoma	2 (22%)	1(17%)	1 (33%)
Focal nodular hyperplasia	2 (22%)	2 (33%)	0 (0%)
Liver hemangioma	2 (22%)	1(17%)	1(33%)
Angiomyolipoma	1 (11%)	0 (0%)	1(33%)
**LIVER RESECTION EXTENT**			
One segment	2 (22%)	1 (17%)	1 (33%)
Two segments	4 (44%)	3 (50%)	1 (33%)
Three segments	2 (22%)	2 (33%)	0
Four segments	1 (11%)	0	1 (33%)
Duration of surgery, min	339.4 ± 230.5	276 ± 96	318 ± 162
Blood loss, ml	1378 ± 1063	1216 ± 1188	1700 ± 866
**HEMODYNAMIC PARAMETERS**			
CVP, cmH_2_O	4.9 ± 1.9	4.9 ± 2	5 ± 1.8
SBP, mmHg	115 ± 7.5	114 ± 4.6	117 ± 13
HR, beats/min	72 ± 17	75 ± 21	66 ± 1.4
**BLOOD PRODUCT TRANSFUSIONS**			
Red blood cells, ml, (*n*)	530 ± 406 (7)	459 ± 442 (4)	672 ± 355 (3)
Freshly frozen plasma, ml, (*n*)	479 ± 338 (7)	459 ± 403 (4)	518 ± 218 (3)
Cryoprecipitate, ml, (*n*)	480 ± 28 (2)	480 ± 28 (2)	0 (0)

*Data presented as mean ± SD, number (n). COPD, chronic obstructive pulmonary disease; CVP, central venous pressure; HR, heart rate; kg, kilograms; min, minutes; ml, milliliters; SBP, systolic blood pressure*.

The extent of liver resection was from one to four segments with an average duration of surgery of 339.4 ± 230.5 min (min. 190–max. 910 min). The Pringle maneuver lasted from 20 to 25 min (mean 20.5 min). Mean CVP was 4.9 ± 1.9 cm H_2_O, ranging from 2.5 to 8 cm H_2_O during surgery indicating relative hypovolemia. Mean systolic blood pressure was 115.2 ± 7.5 mmHg and mean heart rate was 72 ± 17 beats/min.

Five patients had significant bleeding during surgery, each of them lost more than 1,000 ml of blood. Average blood loss was 1,378 ± 1,063 ml. Seven patients received transfusions of packed red blood cells and fresh frozen plasma, whereas only two of them required fibrinogen substitution with cryoprecipitate (Table 1).

Before surgery, the patients had the following mean blood cell counts: white blood cells 7.30 ± 1.98 × 10^9^/L, red blood cells 4.6 ± 0.33 × 10^12^/L, and platelets 272 ± 97.9 × 10^9^/L. Hb and Hct were 140.3 ± 15.3 g/L and 42 ± 3.49%, respectively. Standard coagulation parameters were within normal ranges: activated partial thromboplastin time (APTT) 30 ± 2.9 s, prothrombin time (PT) 104 ± 14%, International Normalized Ratio (INR) 0.9 ± 0.07 and fibrinogen plasma concentration 3.6 ± 0.18 g/L.

### Changes in PAI-1 and t-PA plasma concentrations

**Figure 2:**
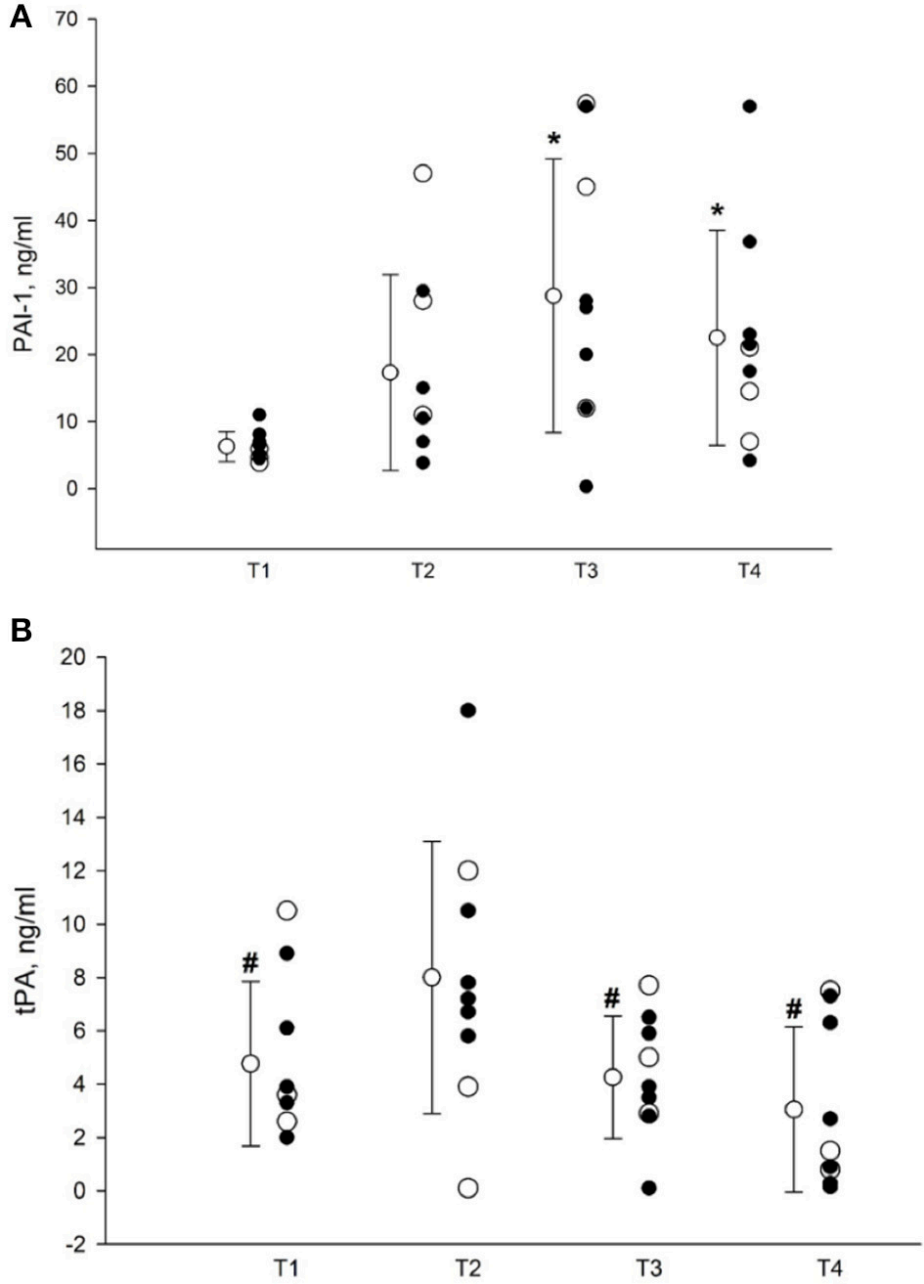
**(A)** Plasma concentrations of PAI-1. T1, before surgery; T2, before Pringle maneuver; T3, at the end of surgery; T4, 24 h postoperatively. Data presented as mean ± SD (*n* = 9). Closed circles represent patients subjected to the Pringle maneuver (*n* = 6); open circles represents subjects in whom the Pringle maneuver was not performed (*n* = 3). *Denotes *p* < 0.05 vs. T1 as assessed by RM ANOVA followed by pairwise multiple comparisons (Student-Newman-Keuls method). PAI-1, plasminogen activator inhibitor, type 1. **(B)** Plasma concentrations of t-PA. T1, before surgery; T2, before Pringle maneuver; T3, at the end of surgery; T4, 24 h postoperatively. Data presented as mean ± SD (*n* = 9). Closed circles represent patients subjected to the Pringle maneuver (*n* = 6); open circles represents subjects in whom the Pringle maneuver was not performed (*n* = 3). ^#^Denotes *p* < 0.05 vs. corresponding T2 value, as assessed by RM ANOVA followed by pairwise multiple comparisons (Student-Newman-Keuls method). t-PA, tissue plasminogen activator.

As shown in Figure 2A, plasma concentration of PAI-1 (*n* = 9) increased from 6.25 ± 2.24 ng/ml at T1 through 17.30 ± 14.59 ng/ml during surgery before start of the Pringle maneuver at T2 and reached its peak concentration of 28.74 ± 20.41 ng/ml (*p* = 0.007) at T3. At T4, PAI-1 plasma level decreased to 22.5 ± 16.2 ng/ml, but still remained elevated in comparison with T1 (*p* = 0.04).

Correspondingly, as depicted in Figure 2B, the plasma concentration of t-PA (*n* = 9) rose from 4.76 ± 3.08 at T1 to 8.00 ± 5.1 at T2, and decreased to 4.25 ± 2.29 ng/ml and 3.04 ± 3.1 at T3 and T4, respectively. Thus, the t-PA level at T2 differed significantly from those determined at T1, T3, and T4 (*p* < 0.004).

### Sample size calculations

We used the intragroup changes in PAI-1 concentrations between T1 and T3, and the differences in t-PA level between T2 and T4 to calculate sample sizes of a future prospective study. Based on mean clinical relevant intragroup differences of PAI-1 and t-PA of 22.5 and 5.00 ng/ml, respectively, we estimated sample sizes of 9 and 13 patients for each variable, respectively. Concerning patients undergoing Pringle maneuver, the least sample size required for obtaining significant intragroup differences in PAI between T1 and both T3 and T4, was 10 patients. Between those who underwent Pringle maneuver at T3 vs. those who did not, mean difference in PAI-1 was 14 ng/ml. Given standard deviations of 19.00 ng/ml and 23.5 ng/ml, respectively, would require 60 and 30 patients in the Pringle and the non-Pringle groups.

### Relationships between PAI-1 plasma concentrations and volumes of transfused blood products

**Figure 3:**
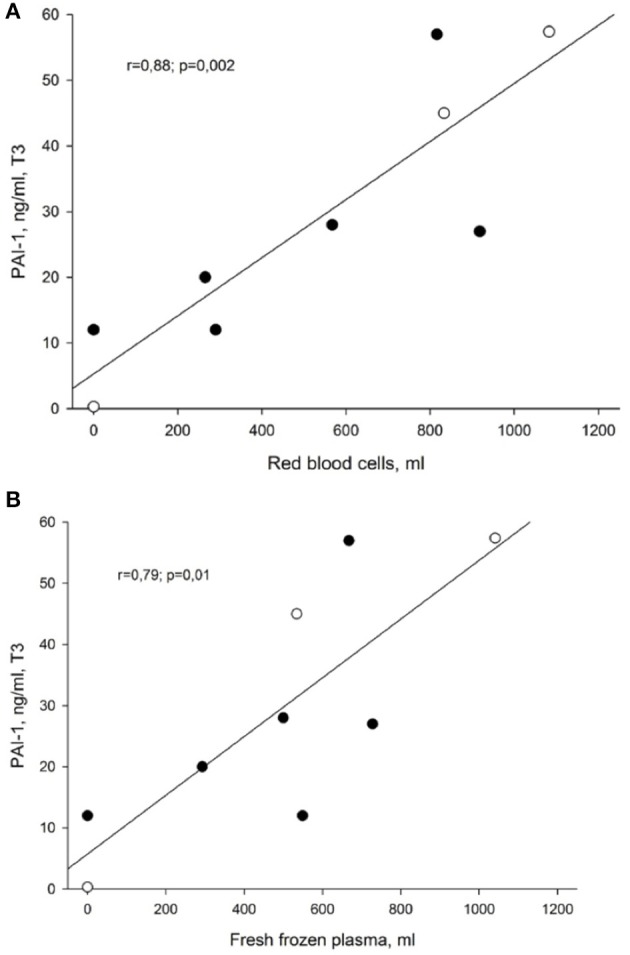
**(A)** Correlation between PAI-1 and transfused volume of red blood cells at the end of liver surgery (T3). Data presented as Pearson correlation coefficient (r) with *p*-value. PAI-1, plasminogen activator inhibitor, type 1. **(B)** Correlation between PAI-1 and transfused volume of fresh frozen plasma at the end of liver surgery (T3). Data presented as Pearson correlation coefficient (r) with *p*-value. PAI-1, plasminogen activator inhibitor, type 1.

Statistically significant correlations were observed at T3 between PAI-1 plasma levels and transfused volumes of fresh frozen plasma and red blood cells. As depicted in Figures 3A,B, the patients who presented with the highest PAI-1 plasma concentrations at T3 also received most transfusions of red blood cells (*r* = 0.9; *p* = 0.002) and fresh frozen plasma (*r* = 0.8; *p* = 0.01). We did not find corresponding relationships between t-PA and transfused volumes of blood products.

### Associations between extent of liver resection and PAI-1 and t-PA plasma concentrations at the end of surgery

Plasma concentrations of PAI-1 increased gradually with the extent of liver resections, albeit without reaching statistical significant difference. At the end of surgery (T3), mean PAI-1 plasma concentration was 13.6 ± 19 ng/ml in patients subjected to resection of one liver segment, and correspondingly 29 ± 19 ng/ml after resection of two segments, and 34.7 ± 32 ng/ml and 45 ng/ml after three and four liver segment resections, respectively.

A similar tendency, was seen when analyzing the corresponding t-PA plasma concentrations. At the end of surgery (T3), t-PA was 3 ± 4.1 ng/ml in patients subjected to resection of a single liver segment, and 4.7 ± 1.3 ng/ml and 5.2 ± 3.5 ng/ml, respectively, in those who underwent two and three segment resections. Surprisingly, we found the lowest t-PA level of 2.9 ng/ml in the patient with four liver segment resections.

### Effects of pringle maneuver on PAI-1 and t-PA

We also compared PAI-1 and t-PA plasma concentrations after surgery at T3 in patients subjected to the Pringle maneuver (*n* = 6) with those in whom the maneuver was not applied (*n* = 3). In those exposed to the Pringle maneuver, PAI-1 plasma concentration increased from 7.00 ± 2.37 ng/ml at T1 to 24.05 ± 19.16 ng/ml (NS) at T3, and further to 26.66 ± 18.17 ng/ml (NS) at T4. In subjects not exposed to the Pringle maneuver, PAI-1 increased significantly within group from 4.76 ± 1.05 ng/ml at T1 to 38.13 ± 23.46 ng/ml at T3 (*p* = 0.043), and decreased to 14 ± 7 ng/ml (NS) at T4.

In patients subjected to the Pringle maneuver, plasma t-PA levels increased from 4.36 ± 2.68 ng/ml at T1 through 9.33 ± 4.53 ng/ml at T2 (*p* = 0.001), and subsequently decreased to 3.78 ± 2.30 at T3 (*p* = 0.011 vs. T2) and to 2.93 ± 3.14 ng/ml (*p* = 0.037 vs. T2) at T4. In patients not undergoing the Pringle maneuver, t-PA fell slightly from 5.56 ± 4.30 ng/ml at T1 to 5.20 ± 2.40 ng/ml at T3 and to 3.26 ± 3.68 ng/ml at T4 (NS). However, we found no intergroup differences in PAI-1 and tPA plasma concentrations depending on whether the Pringle maneuver was used or not.

## Discussion

In patients subjected to liver surgery, we observed increments in fibrinolytic activity, as assessed by changes in plasma concentrations of PAI-1 and t-PA. In nine patients undergoing, at least, one segment resection, we found significant associations after surgery between PAI-1 plasma levels and volumes of transfused red blood cells and fresh frozen plasma. However, we noticed no such associations with t-PA.

The liver is responsible for synthesis and clearance of several hemostatic factors. Increased fibrinolytic activity occurs as a shift in the balance between pro-fibrinolytic (t-PA) and anti-fibrinolytic (PAI-1) factors, as characterized by an increase in global fibrinolytic capacity and a reduction of clot lysis time, particularly in patients with liver cirrhosis (11). Liver surgery on its own right, and the use of the Pringle maneuver with major endothelial damage, can reduce hepatic perfusion, increase hepatocellular dysfunction and promote a hyper-fibrinolytic state (8, 20, 21). Therefore, prophylaxis with anti-fibrinolytic agents are recommended in patients scheduled for liver surgery (22–24). Although, all the patients received a prophylactic dose of 10 mg/kg of tranexamic acid after induction of anesthesia, its modulating effect on the activation of fibrinolysis and bleeding is still questionable.

As a part of our preoperative routines, we evaluated the perioperative morbidity and mortality risks by means of the *Child-Pugh* score, which is widely used to assess the prognosis of patients with liver cirrhosis (25). With a score of less than 6 points, corresponding to a Child–Pugh class A, we predicted the one-year survival rate of our patients to 100% (26, 27). Therefore, we considered the perioperative mortality risk as low, which we also confirmed by observing that no patient died or experienced serious complications during or after the surgery.

It is well documented that the plasma level of t-PA, the main fibrinolytic activator responsible for the cleavage of plasminogen to plasmin, is elevated in patients with chronic liver disease (5). We observed no elevations in t-PA before the surgery, except for in one patient, in whom plasma t-PA was 10.5 ng/ml at T1 increasing to 12.5 ng/ml at T2. The latter patient, who underwent resection of three segments in an operation lasting for more than 6 h, also presented with a blood loss of 2,300 ml, which was the second highest in the group. Another patient, who underwent segment resection, which lasted for more than 3 h, had no increase in t-PA preoperatively, but displayed an elevated concentration of 10.5 ng/ml at T2, which was associated with a blood loss of 2,200 ml. However, we were not able to find any significant correlations between bleeding volumes and t-PA plasma concentrations in the patient population as a whole.

Trauma and tissue hypoperfusion might trigger traumatic coagulopathy. Following trauma and tissue hypoperfusion, thrombin activates protein C on the surface of endothelial cells via the endothelial protein C receptor and the membrane-bound glycoprotein thrombomodulin, forming the thrombin–thrombomodulin complex. Low plasma protein C levels and high plasma thrombomodulin levels are associated with increased mortality (28). Vascular endothelial cells, smooth muscle cells, hepatocytes, and platelets produce PAI-1, an acute phase protein and major inhibitor of t-PA, in response to sepsis, various cytokines, surgery and trauma (29). After activation of protein C, PAI-1 might fall below normal level (7). However, in our patients, mean plasma concentration of PAI-1 before the surgery was within the normal range (5–20 ng/ml). Moreover, as depicted in Figures 3A,B, patients who had higher PAI-1 plasma concentrations at T3, also received significantly more fresh frozen plasma and red blood cell transfusions. Presently, it is not known, whether a low plasma PAI-1 level is predictive of clinical outcome after a major blood loss such as during haemorhagic shock.

We observed the most rapid increments in marker plasma concentrations at the beginning of surgery. PAI-1 increased nearly three-fold when comparing the concentration determined at T1 with that found at T2 and almost 5-fold, as compared with that at the end of surgery (T3). We interpret these changes as secondary to activation of the fibrinolytic system in the presence of an insufficient inhibition. Increments in the concentration of t-PA were rather modest, as assessed by an elevation of 68% at T2 as compared with that at T1. Rapid increments in these marker concentrations at the start of surgery might be explained by endothelial and surgical tissue damages, as described by recent investigators (1, 30).

Our findings are consistent with those of Illig and co-authors, who studied primary fibrinolysis during supraceliac clamping of the aorta (31). The latter included 10 consecutive patients and compared them with eight controls, who underwent infra-renal clamping of the aorta during abdominal aortic reconstruction surgery. Supraceliac clamping rapidly induced a primary fibrinolytic state characterized by increased circulating t-PA and reduced alpha 2-antiplasmin. The investigators interpreted their findings as secondary to hepatic hypoperfusion. In the present study, the Pringle maneuver was associated with a significant increase in t-PA plasma concentration after the surgery. This observation is consistent with findings reported by other recent researchers (32–34). The latter investigators demonstrated that the Pringle maneuver might intensify hepatic ischemia, reperfusion injury, and blood vessel trauma. Consequently, it promotes t-PA secretion from endothelial cells in response to a variety of noxious stimuli such as exposure to endotoxin, arterial ischemia and venous occlusion (27, 35). According to recent recommendations, the Pringle maneuver should be performed intermittently limited to a duration of no more than 1 h (36). Therefore, we performed the Pringle maneuver within a time limit of 30 min. However, we were not able to display a correlation between fibrinolytic markers and time of clamping.

According to recent studies, mean blood loss in otherwise healthy patients undergoing liver surgery is ~600–900 ml, which is less than in our patients. We found a blood loss ranging from 100 to 3,000 ml. This discrepancy might be due to comorbidities and a high age difference between the patients, combined with the fact that liver resection surgery in Latvia was implemented rather recently (30, 37).

After analyzing the influence of the extent of tissue resection on fibrinolytic activity, we observed that in patients with more extensive liver resections, PAI-1 plasma concentrations increased before application of the Pringle maneuver and at the end of surgery, which might be explained by a larger surgical tissue trauma. Thus, apparently, the extent of liver surgery influenced t-PA plasma levels to a lesser degree. According to recent literature, fibrinolytic activity might increase before surgery in patients with chronic liver diseases, as documented by elevated t-PA plasma levels (30).

Surprisingly, the patient with a four segment liver resection had a low t-PA plasma level at the end of surgery and 24 h postoperatively. Most likely, this is explained by a decrease in hepatic clearance and low t-PA plasma levels already before the surgery. Low plasma levels of t-PA at baseline could be due to high age and such co-morbidities as general atherosclerosis, arterial hypertension, type 1 diabetes and metabolic syndrome. These co-morbidities were frequently associated with reduced levels of t-PA and elevated PAI-1 concentrations (38). However, in the present study, we observed lower t-PA and higher PAI-1 levels already before the surgery.

We admit as a major limitation that the study is undersized to detect significant intragroup differences in t-PA and intergroup differences in both PAI-1 and t-PA between patients who underwent the Pringle maneuver and those who did not. The reason for this shortage is that we introduced systematic liver surgery rather recently. Moreover, because of limited experience both with anesthetic and surgical management of patients undergoing liver surgery we intentionally avoided to include a control group not given the prophylactic standard dose of tranexamic acid. This kind of surgery requires multidisciplinary treatment and application of demanding techniques, which in addition to patient factors explain both the small number of patients included, and the relatively high blood losses.

Another limitation is the heterogenic group of patients studied. Some of them had different co-morbidities such as diabetes, primary arterial hypertension that might affect baseline fibrinolytic activity. We do not know to what degree fibrinolytic activation due to surgical trauma and blood loss occur in these patients in comparison with those without such cardiovascular co-morbidities.

Unfortunatelly, we did not meassure changes in activated protein C plasma levels in parallel with changes in PAI-1 concentrations. Because of low sample sizes and no deaths in our pilot study, we were not able to analyze the impact of changes in plasma concentrations of PAI-1 on mortality or other measures of clinical outcome. However, we found corelations between PAI-1 level after surgery and transfused volumes of red blood cells and fresh frozen plasma.Considering the limited number of patients in this pilot study and their initial *Child-Pugh* scores, we should be cautious to extrapolate the results to a larger cohort of patients scheduled for liver surgery. In accordance with our sample size calculations, we expect that a future prospective multicenter study of at least 90 patients planned for liver surgery, which also includes the Pringle maneuver, might shed more light on fibrinolysis as a cause of perioperative bleeding.

## Conclusions

During liver resection surgery, fibrinolytic system activation increases as experienced by a significant release of PAI-1, in parallel with increments in t-PA, especially at the start of surgery. Application of the Pringle maneuver was associated with a trend toward more pronounced activation of fibrinolysis. Although, we found no associations between fibrinolytic markers and bleeding volumes, higher plasma concentrations of PAI-1 correlated highly with the volumes of transfused blood products. Further analysis including more patients is necessary to confirm the augmentation of fibrinolysis and its correlation with bleeding volumes during liver surgery.

## Author contributions

All authors have contributed equally to the design of the study, to analysis and interpretation of results, and drafting or revising the manuscript. AgO, JN, and AK conceived the study. AgO included patients into the study and got agreement of participation, as well as continued to collect the data. JN was responsible for administering anesthesia in a standardized manner to all the included patients and ArO and JG performed the surgery. IV, AK, and LV performed data analysis and interpreted the results. AgO and LB drafted and revised the manuscript. All authors read and approved the final manuscript.

### Conflict of interest statement

The authors declare that the research was conducted in the absence of any commercial or financial relationships that could be construed as a potential conflict of interest.
